# Ansa pancreatica: a rare cause of acute recurrent episode in chronic pancreatitis

**DOI:** 10.1259/bjrcr.20200044

**Published:** 2020-09-30

**Authors:** Hind Guerroum, Amal Rami, Mariam Kassimi, Jihane Habi, Rahmouni Imane, Nabil Chikhaoui, Fadwa Rouibaa, Mohamed Mahi

**Affiliations:** 1 Department of Radiology, Faculty of Medicine, Mohammed VI University of Health Sciences/Cheikh Khalifa International University Hospital, Casablanca, Morocco; 2 Department of Gastroenterology, Faculty of Medicine, Mohammed VI University of Health Sciences/Cheikh Khalifa International University Hospital, Casablanca, Morocco

## Abstract

Ansa pancreatica is a rare anatomic variation of pancreatic ducts. It is a predisposing factor of recurrent pancreatitis. In this case report, we describe a case of a 24-year-old male suffering from an ansa pancreatica with a non-patent major papilla, diagnosed on magnetic resonance cholangiopancreatography (MRCP).The ansa pancreatica was revealed by an episode of acute pancreatitis attacks in chronic pancreatitis. Endoscopic retrograde cholangiopancreatography (ERCP) confirmed important abrupt dilation in the main pancreatic duct with an ansa loop in the pancreatic duct in the head of the pancreas, and a sphincterotomy of the minor papilla was performed. The procedure was difficult and the placement of a long-term pancreatic stent during the ERCP was impossible, thus a surgical pancreatico-jejunostomy was proposed as a treatment of an ansa pancreatica with a non-patent major papilla.

## Introduction

Chronic pancreatitis is defined as a chronic inflammation of the pancreas. At the early stages, its clinical presentation consists of recurrent episodes of acute pancreatitis. Alcohol is the most common cause of chronic pancreatitis.^1,2^ Pancreatic ductal anatomical variations play also a role in causing recurrent pancreatitis.^3^ Ansa pancreatica is a rare ductal anomaly where the duct of Santorini forms a loop or S-shaped as it joins the duct of Wirsung, which reduces the flow of pancreatic juice.^2,3^

## Case report

A 24-year-old male with several previous episodes of acute pancreatitis of unknown etiology, presented to the gastroenterology clinic with severe epigastric pain, serum lipase was greater than four times the upper limit of normal, and the abdominal ultrasound found no gallstones or important biliary dilatation.

**Figure 1.**
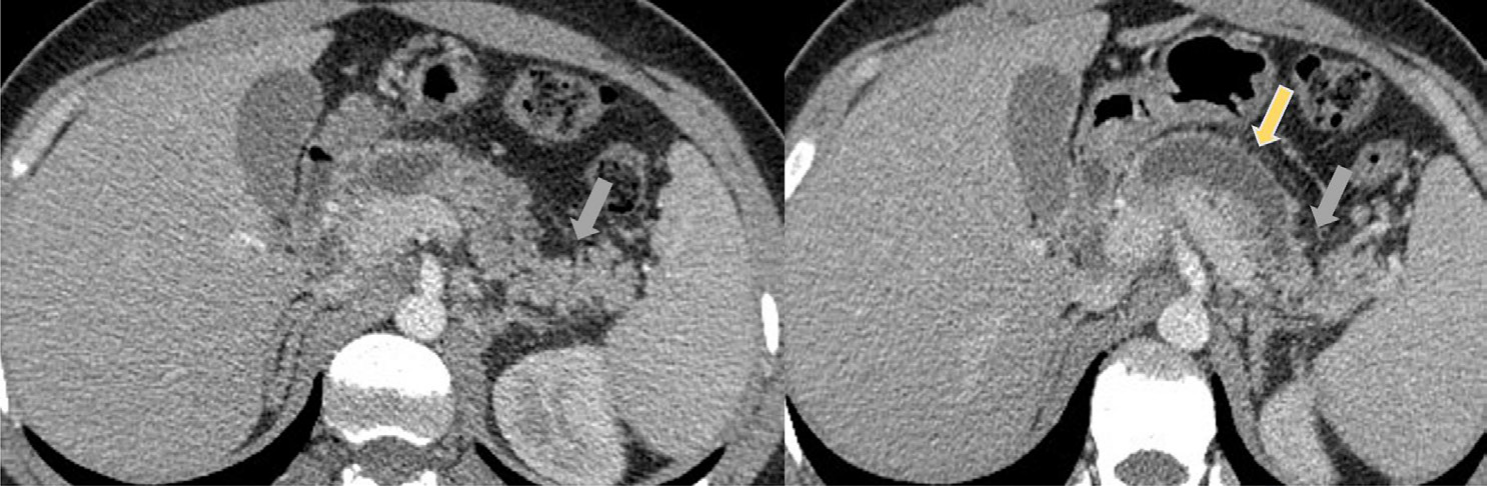
Contrast-Enhanced Computerised Tomography (CECT) showed a mild pancreatitis (Atlanta Classification (2012)) with an abrupt dilatation of pancreatic duct to 16mm (yellow arrow), and atrophy of the surrounding parenchyma as a sign of chronic pancreatitis (grey arrow).

Contrast-enhanced computerized tomography (CECT) 3 days later showed mild pancreatitis (Atlanta Classification (2012)) with an abrupt dilatation of pancreatic duct to 16 mm (yellow arrow), and atrophy of the surrounding parenchyma as a sign of chronic pancreatitis (grey arrow) (Figure 1).

The patient received standard treatment which is a brief period of bowel rest and adequate hydration with i.v. fluids, with significant improvement in symptoms.

There were no other medical history or medication usage, the patient was not known alcoholic and triglycerides were within normal limits. Further tests showed no exocrine insufficiency.

**Figure 2.**
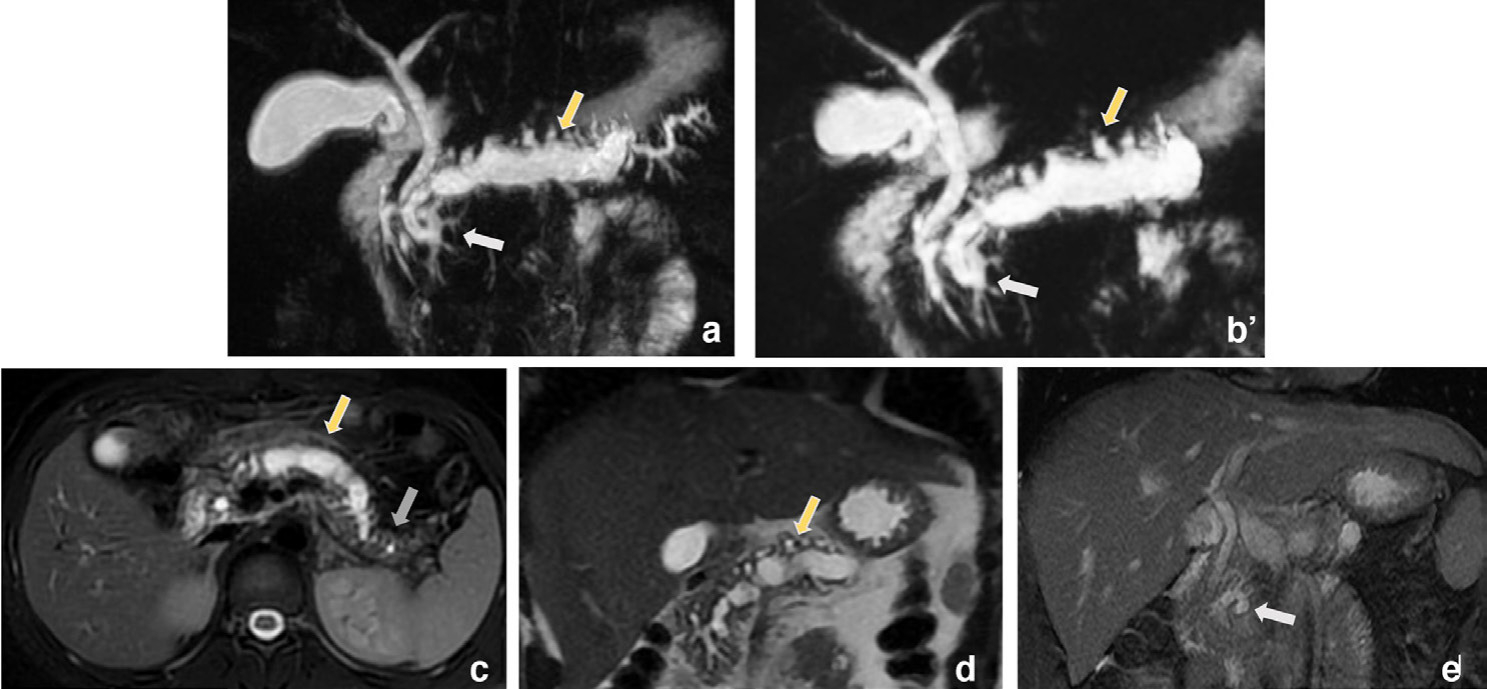
Magnetic resonance cholangiopancreatography (MRCP) showed a dilatation of main pancreatic duct to 16mm (yellow arrow) with atrophy (grey arrow) of the surrounding parenchyma as a sign of chronic pancreatitis, and an ansa loop in the duct of Santorini (white arrow) in the head of the pancreas as it joins the duct of Wirsung revealing an ansa pancreatica with a non-patent major papilla.

A magnetic resonance cholangiopancreatography (MRCP) was performed and showed a dilatation of main pancreatic duct to 16 mm (yellow arrow) with atrophy (grey arrow) of the surrounding parenchyma as a sign of chronic pancreatitis, and an ansa loop in the duct of Santorini (white arrow) in the head of the pancreas as it joins the duct of Wirsung revealing an ansa pancreatica with a non-patent major papilla. (Figure 2)

Endoscopic retrograde cholangiopancreatography (ERCP) confirmed important dilation in the main pancreatic duct with an ansa loop in the duct of Santorini as it joins the duct of Wirsung in the head of the pancreas, with a non-patent major papilla. A sphincterotomy of the minor papilla was performed. (Images are not available).

The procedure was difficult and the placement of a long-term pancreatic stent during the ERCP was impossible.

A surgical pancreatico-jejunostomy was proposed as a treatment of an ansa pancreatica with a non-patent major papilla.

## Discussion

The normal pancreatic ductal system includes the ventral duct known as Wirsung’s duct and the dorsal duct also known as Santorini’s duct, they fuse in the head of the pancreas. Most of the drainage occurs through the duct of Wirsung to the major papilla while a small portion of pancreatic secretion is drained by the duct of Santorini through the minor papilla.^2,4^

During embryological development, the merge of two ducts occurs around 6 to 8 weeks of gestation,^2,4^ however abnormal ductal fusion may cause many anatomic variations of pancreatic ducts such as ansa pancreatica.

In 1961, Dawson and Langman were the first to describe ansa pancreatica in literature as an obliteration of the Santorini’s duct where it joins the Wirsung’s duct.^5^ The merging of the two ducts is substituted by a loop connecting the inferior branch of the dorsal duct and the inferior branch of the ventral duct. The communication into the duodenum via either minor or major papilla was not always patent, thereby the drainage of pancreatic secretions was not allowed freely.^5,6^

Adibelli et al established a study in turkey, including 1158 patients who underwent MRCP in their institution. They conclude that only 1.2% were defined as ansa pancreatica.^7^ Another Japanese study by Hayashi et al including 587 patients who underwent MRCP also showed that 0.85% of patients had ansa pancreatica.^8^ Sill the prevalence of ansa pancreatica is not well studied in the literature due to its rarity.

Our patient presented a ductal variant of ansa pancreatica were Santorini’s duct emits a loop as it joins the Wirsung’s duct but with a non-patent major papilla.

Ansa pancreatica is a predisposing factor to recurrent pancreatitis because of the reduced flow of pancreatic secretion caused by the sloping angle connecting the arched duct from the duct of wirsung and the accessory duct. Especially for patients in alcoholism and functional stenosis of the sphincter of Oddi.^3^ According to Hayashi et al, the risk of acute and recurrent pancreatitis is higher in patients with ansa pancreatica (20%) versus the ones without it (0.52%).^8^

The diagnosis of chronic pancreatitis includes “recurrent bouts of pain with or without ≥3 fold the normal upper limit of amylase or lipase levels” and at least on two of the following criteria: “Radiological evidence comprising strictures and dilatation inside branches and/or the main pancreatic duct and/or intraductal and/or parenchymal pancreatic calcifications by contrast-enhanced CT and magnetic resonance cholangiopancreatography, and/or Histological proof of chronic pancreatitis from biopsy samples undertaken by endoscopic ultrasonography or from a surgically resected specimen”.^1^ Nonetheless if the histological and/or radiological evidence is absent, the diagnosis would be recurrent acute pancreatitis instead of chronic pancreatic.^1^

According to the European gastroenterology (UEG), endoscopic ultrasound (EUS), MRI, and CT are the best-imaging tools to diagnose chronic pancreatitis. The ERCP is an invasive procedure and thus not considered as a diagnostic method.^9^

The MRI is perfectly able to set the etiological diagnosis and to detect the ansa loop on 3D-T2W images.^2^

In our case, besides recurrent episodes of abdominal pain and biochemical evidence, chronic pancreatitis was diagnosed on abdominal CT and MRCP. The diagnosis of ansa pancreatica was based on the MRCP. (Figure 2)

The treatment of chronic pancreatitis consists of appeasing the pain, and supplying the exocrine and endocrine insufficiency, in order to prevent the complications and the progression of the disease.^1^

According to Ana Dugic et al^10^ , the role of pancreatic ductal variations in the insufficiency of pancreatic exocrine function is still controversial.^1^ Our patient’s pancreatic exocrine function was conserved.

Specific treatment of pancreatitis in ansa pancreatica patients is not well described in the literature^1^ Justin S Kosirog et al performed a sphincterotomy of the major papilla and placed 5 French by 3 cm plastic stent in the PD (pancreatic duct) downstream to the ansa loop.^3^

Our patient received standard treatment which is a brief period of bowel rest and adequate hydration with i.v. fluids, with significant improvement in symptoms.

ERCP confirmed important dilation in the main pancreatic duct with an ansa loop in the duct of Santorini as it joins the duct of Wirsung in the head of the pancreas, and a sphincterotomy of the minor papilla was performed. The procedure was difficult and the placement of a long-term pancreatic stent during the ERCP was impossible.

A surgical pancreatico-jejunostomy was proposed as a treatment of an ansa pancreatica with a non-patent major papilla.

## Conclusion

Ansa pancreatica is a rare anatomic variant of the pancreatic ducts accounting for only about 1% in the literature.^2^ Its presence should be investigated in cases of idiopathic recurrent acute pancreatitis, in order to prevent a chronic pancreatitis.

## Learning points

Ansa pancreatica is a rare anatomic variation of pancreatic ducts.

The presence of ansa pancreatica should be investigated in cases of idiopathic recurrent acute pancreatitis, in order to prevent a chronic pancreatitis.

MRCP can diagnose ansa pancreatica.
